# Streptococcal M protein promotes IL-10 production by cGAS-independent activation of the STING signaling pathway

**DOI:** 10.1371/journal.ppat.1006969

**Published:** 2018-03-26

**Authors:** Elin Movert, Julia Lienard, Christine Valfridsson, Therése Nordström, Bengt Johansson-Lindbom, Fredric Carlsson

**Affiliations:** 1 Department of Experimental Medical Science, Section for Immunology, Lund University, Lund, Sweden; 2 Department of Laboratory Medicine, Section for Medical Microbiology, Lund University, Sweden; 3 Department of Biology, Section for Molecular Cell Biology, Lund University, Lund, Sweden; Children's Hospital Boston, UNITED STATES

## Abstract

From an evolutionary point of view a pathogen might benefit from regulating the inflammatory response, both in order to facilitate establishment of colonization and to avoid life-threatening host manifestations, such as septic shock. In agreement with this notion *Streptococcus pyogenes* exploits type I IFN-signaling to limit detrimental inflammation in infected mice, but the host-pathogen interactions and mechanisms responsible for induction of the type I IFN response have remained unknown. Here we used a macrophage infection model and report that *S*. *pyogenes* induces anti-inflammatory IL-10 in an M protein-dependent manner, a function that was mapped to the B- and C-repeat regions of the M5 protein. Intriguingly, IL-10 was produced downstream of type I IFN-signaling, and production of type I IFN occurred via M protein-dependent activation of the STING signaling pathway. Activation of STING was independent of the cytosolic double stranded DNA sensor cGAS, and infection did not induce detectable release into the cytosol of either mitochondrial, nuclear or bacterial DNA–indicating DNA-independent activation of the STING pathway in *S*. *pyogenes* infected macrophages. These findings provide mechanistic insight concerning how *S*. *pyogenes* induces the type I IFN response and identify a previously unrecognized macrophage-modulating role for the streptococcal M protein that may contribute to curb the inflammatory response to infection.

## Author summary

The biological role of type I IFN in bacterial infection may differ dependent on etiological agent. With regard to *S*. *pyogenes* infection the type I IFN response has been shown to protect the host by limiting detrimental inflammation without impacting on bacterial load, suggesting a selective pressure on *S*. *pyogenes* to induce type I IFN to promote host survival. Production of type I IFN in *S*. *pyogenes* infected macrophages occurs in a STING-, TBK1- and IRF3-dependent manner. Still, the host-pathogen interactions and mechanisms responsible for activation of the STING signaling pathway have remained unknown. We used a macrophage infection model and show that the *S*. *pyogenes* M protein promotes the production of anti-inflammatory IL-10. Remarkably, IL-10 was produced downstream of type I IFN-signaling, and production of type I IFN occurred via an active and M protein-dependent mechanism that required STING but that was independent of cGAS and cytosolic sensing of DNA. These findings advance our understanding of how *S*. *pyogenes* induces the type I IFN response and describe a conceptually novel macrophage-modulating role for the streptococcal M protein that may contribute to limit the inflammatory response to infection.

## Introduction

*Streptococcus pyogenes* is a Gram-positive human pathogen causing a wide spectrum of clinical manifestations, ranging from mild infections of the skin or mucosal surfaces to invasive and life-threatening conditions such as necrotizing fasciitis and toxic shock syndrome [1]. The ability of *S*. *pyogenes* to spread and persist within the human population can be ascribed to its virulence factors, where the surface-anchored M protein plays a critical role in colonization and virulence [2, 3]. The M protein occurs in >200 types (M or *emm* types) and is best known for inhibiting complement deposition onto the bacterial surface to prevent phagocytosis by neutrophils, enabling rapid growth in human blood, and for giving rise to type specific immunity [2, 3].

Macrophages exhibit plasticity and may polarize into functionally distinct subsets dependent on environmental cues, where classically activated (M1) macrophages are bactericidal and produce proinflammatory cytokines such as IL-12, TNFα and IL-6 [4]. Under certain conditions a spectrum of alternatively activated (M2) macrophages may develop, including regulatory macrophages that are characterized by production of the immunosuppressive cytokine IL-10 [4]. *In vivo* studies have demonstrated a key role for macrophages in controlling *S*. *pyogenes* infection [5, 6]. However, while macrophages contribute to eliminate *S*. *pyogenes*, it is also thought that uncontrolled activation and release of proinflammatory cytokines from these cells may promote excessive and host-detrimental inflammation, including septic shock [5, 7], suggesting an evolutionary pressure on the bacterium to modulate macrophage-mediated inflammation in order to preserve its host. Consistent with this notion gene expression analyses of infected macrophages have demonstrated that *S*. *pyogenes* drives an atypical activation program with characteristics of both M1 and M2, notably including production of IL-10 [8].

Type I interferons (IFN; IFNα/β) are most well known for anti-viral activities associated with Th1/M1-type responses. However, the biological role of type I IFN may differ dependent on context and has been linked to IL-10 production in various inflammatory settings, such as inflammatory bowel disease [9] and mycobacterial infection [10–12]. In an invasive mouse model of *S*. *pyogenes* infection type I IFN-signaling protects the animals by limiting host-detrimental inflammation, at least in part by suppressing neutrophil influx [13] and IL-1β production [14]. Production of type I IFN in *S*. *pyogenes* infected macrophages occurs in a stimulator of interferon genes (STING)-, TANK-binding kinase 1 (TBK1)- and interferon regulatory factor 3 (IRF3)-dependent manner [13]. Still, the host-pathogen interactions and mechanisms responsible for activation of the STING signaling pathway have remained unknown.

Here we uncover a functional role for the M protein in regulating macrophage cell biology–providing mechanistic insight into how *S*. *pyogenes* activates the STING pathway and type I IFN production, and demonstrating a causal link between this process and generation of the IL-10 producing macrophage phenotype.

## Results

### *S*. *pyogenes* promotes an IL-10 producing macrophage phenotype in an M protein-dependent manner

**Fig 1:**
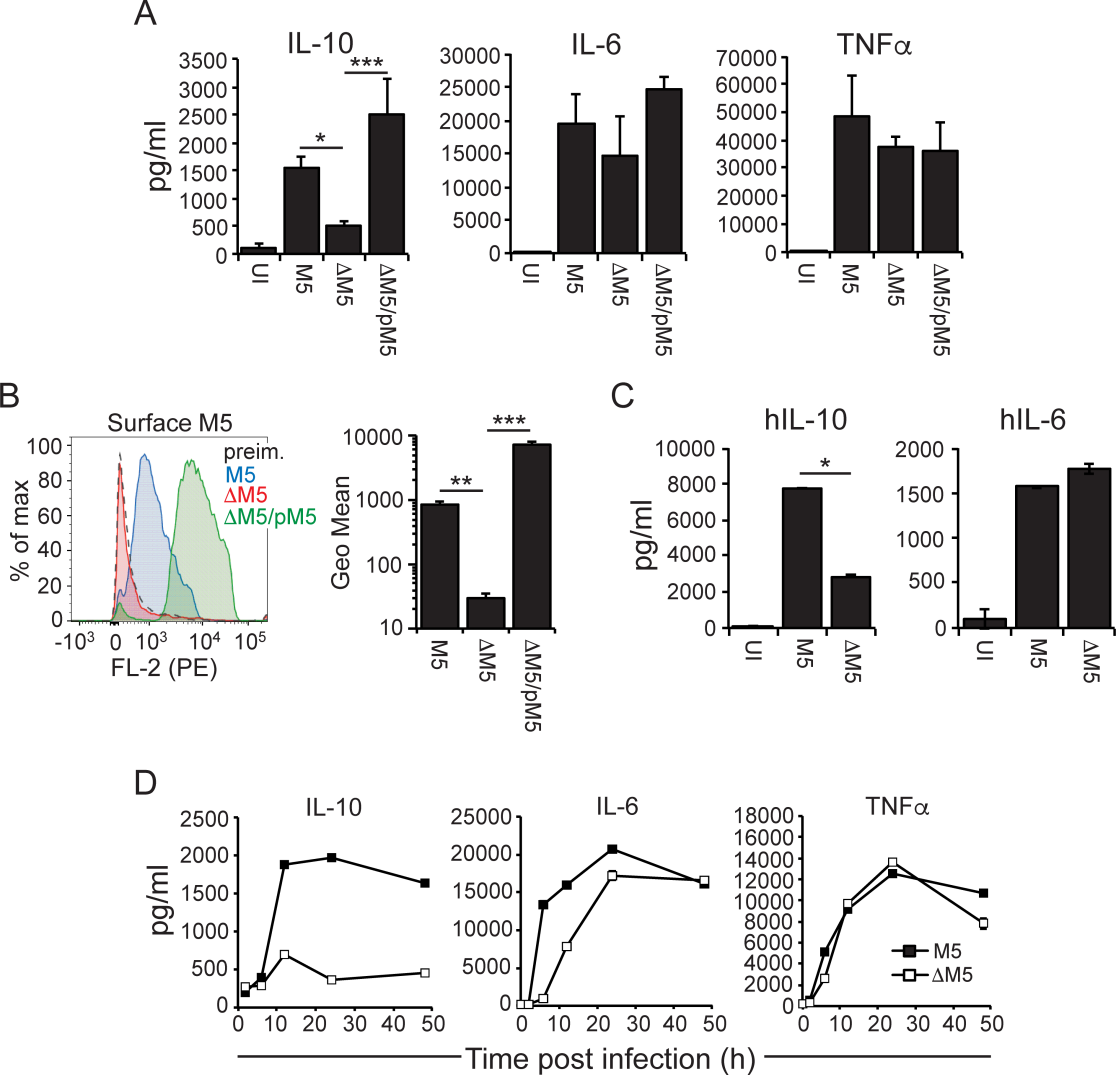
*S*. *pyogenes* promotes IL-10 secretion from infected macrophages in an M5 protein-dependent manner. **A)** Wild type C57BL/6 (B6) bone marrow-derived macrophages were infected with wild type M5, ΔM5 or ΔM5/pM5 *S*. *pyogenes* or uninfected (UI), as indicated. Culture supernatants were collected 24 hours post infection (hpi) and assayed for indicated cytokines. Results shown (mean and SD; *n* = 3 per group) are representative of three independent experiments. **B)** Indicated bacterial strains were analyzed for reactivity with a rabbit antiserum against the N-terminal region of the M5 protein by flow cytometry. As control we analyzed binding to M5 bacteria of a preimmune serum from the same rabbit. Left panel: Shown is a representative histogram. Right panel: Geometrical mean (mean and SD) of three independent experiments. **C)** Human monocyte-derived macrophages were infected as indicated. Culture supernatants were collected 24 hpi and assayed for indicated cytokines. Results shown (mean and SD; *n* = 3 per group) are representative of two independent experiments. **D)** Kinetic analysis of cytokine secretion from B6 macrophages infected as indicated. Results shown (mean; *n* = 2 per group) are representative of two independent experiments. ANOVA (*<0.033; **<0.002; ***<0.001).

To explore a potential role for the M protein in regulating macrophage responses, we infected bone marrow-derived C57BL/6 (B6) macrophages with the wild type serotype M5 Manfredo strain (M5) or its isogenic M protein-deficient mutant (ΔM5). While the secretion of proinflammatory cytokines IL-6 and TNFα was similar in both infections, the output of regulatory IL-10 was significantly higher in wild type infection, indicating that the M5 protein promotes secretion of IL-10 (**Figs 1A and S1**). Transcomplementation of the ΔM5 mutant with the *emm5* gene (ΔM5/pM5) restored ability to drive IL-10, confirming that the low level of IL-10 secretion from ΔM5 infected macrophages was specifically due to lack of M protein (**Fig 1A**). Flow cytometry analysis using an antiserum against the N-terminal region of the M5 protein confirmed that M5 and ΔM5/pM5, but not ΔM5, bacteria expressed the M5 protein on their surface (**Fig 1B**); the higher density of surface M5 protein on ΔM5/pM5 as compared to wild type bacteria is likely due to increased expression of the *emm5* gene when encoded on the plasmid. Infection of human monocyte-derived macrophages similarly demonstrated M5 protein-dependent secretion of IL-10 (**Fig 1C)**, indicating that the M5 protein promotes IL-10 secretion from infected macrophages of both human and mouse origin. Kinetic analyses of cytokine secretion from infected B6 macrophages further established a key role for the M5 protein in driving secretion of IL-10, which reached peak levels at 12 hours post infection (**Fig 1D**). Similar analyses of IL-6 and TNFα suggested that IL-6 secretion was delayed in the absence of M5 protein, but that both of these cytokines ultimately reached similar levels in wild type and ΔM5 infected macrophages (**Fig 1D**).

### The B- and C-repeat regions of the M5 protein are required to induce IL-10 secretion

**Fig 2:**
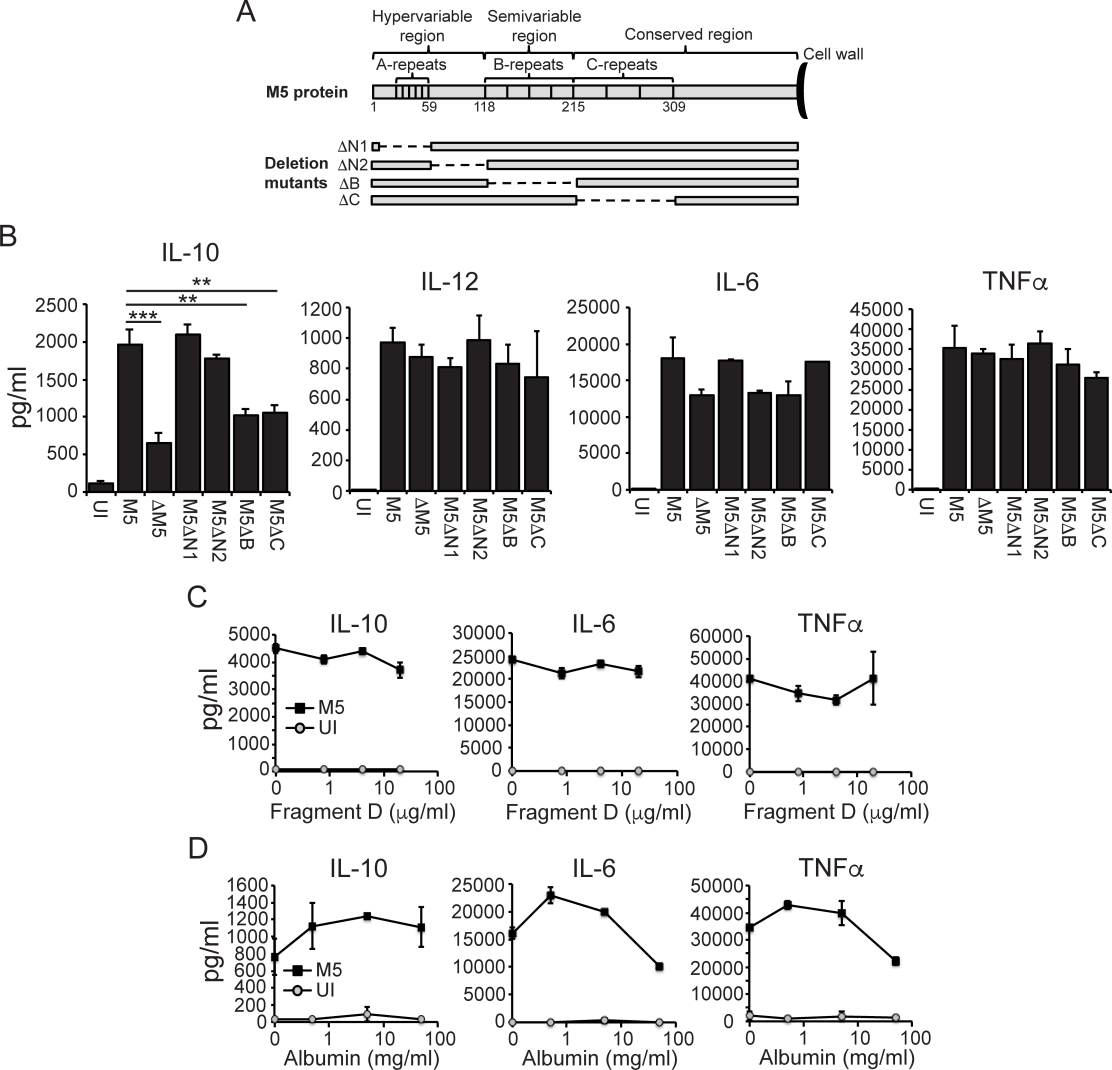
The B- and C-repeat regions of the M5 protein are required to induce IL-10 secretion. **A)** Schematic representation of the M5 protein protruding from the bacterial cell wall, and of the in-frame internal deletion mutants used. **B)** B6 macrophages were infected as indicated, and culture supernatants were collected 24 hpi and assayed for indicated cytokines. Results shown (mean and SD; *n* = 3 per group) are representative of three independent experiments. **C and D)** B6 macrophages were infected with wild type M5 bacteria in the presence of titrated amounts (final concentration indicated) of Fragment D or albumin, as indicated. Culture supernatants were collected 24 hpi and assayed for indicated cytokines. Results shown (mean ± SD; *n* = 3 per group) are representative of three independent experiments. ANOVA (*<0.033; **<0.002; ***<0.001).

The M5 protein is a dimeric coiled-coil protein containing distinct repeat regions–denoted A-, B- and C-repeats–that is covalently linked to the bacterial cell wall via the C-terminal region, with its hypervariable N-terminal part protruding from the bacterial surface (**Fig 2A**) [2].

In an attempt to map the ability of the M protein to drive IL-10 secretion we took advantage of four well-characterized in-frame internal deletion mutants expressing M5 proteins lacking specific regions (**Fig 2A**) [15, 16]. Infection with M5ΔN1 and M5ΔN2 generated a cytokine output similar to that of wild type M5 bacteria (**Fig 2B**). In contrast, macrophages infected with the M5ΔB and M5ΔC mutants secreted significantly reduced levels of IL-10 while the secretion of IL-12, IL-6 and TNFα was unaffected (**Fig 2B**), indicating that M5 protein-dependent secretion of IL-10 requires the B- and C-repeat regions. The M5ΔB and M5ΔC mutants were internalized into macrophages similarly to wild type bacteria (**S2 Fig**), suggesting that M5 protein-mediated induction of IL-10 is not explained by differential uptake [17].

As the B- and C-repeat regions bind fibrinogen and albumin, respectively [2], we considered if these ligand-interactions might influence the ability of the M protein to drive IL-10. Because addition of fibrinogen causes extensive clumping of *S*. *pyogenes* we used fibrinogen fragment D, which includes the M protein-binding site in fibrinogen (**S3A and S3B Fig**) [18] as well as the complement-inhibitory function of bacteria-bound fibrinogen (**S3C Fig**) [15]. Titration of fragment D, covering the fibrinogen concentration (~1–2 μg/ml) found in secretions on inflamed mucosal surfaces [19], did not significantly affect IL-10 secretion from wild type infected macrophages (**Fig 2C**). Addition of higher concentrations of fragment D caused detachment of macrophages and was therefore not analyzed. IL-10 secretion was similarly unaffected by addition of albumin (**Fig 2D**); of note, we have previously shown that addition of albumin functionally competes with binding of antibodies directed against the C-repeat region [16]. Collectively these findings suggest that interaction between the B- and C-repeat regions and their known plasma protein ligands does not affect the ability of the M5 protein to trigger IL-10 production.

### Selective and M5 protein-dependent induction of IL-10 is not explained by regulation of MyD88 activation

Because MyD88-signaling may regulate the inflammatory response in *S*. *pyogenes* infection [20] we compared the cytokine response to wild type M5 bacteria in wild type (B6) and MyD88-deficient (MyD88-KO) macrophages.

**Fig 3:**
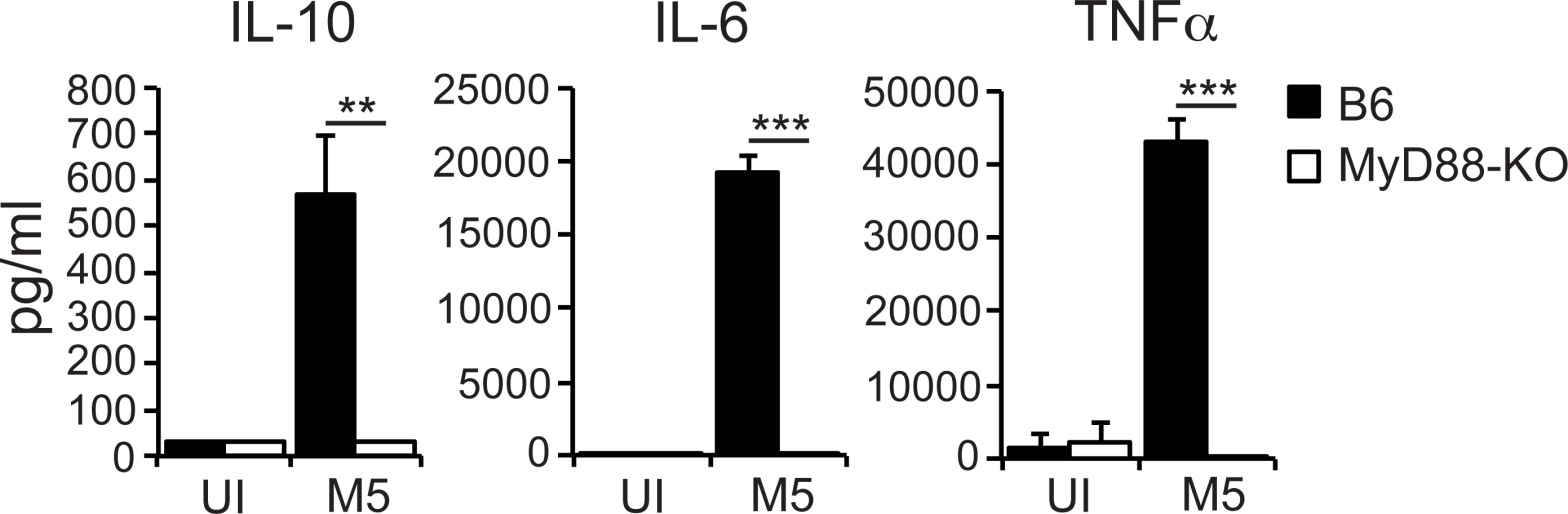
Selective induction of IL-10 is not explained by M5 protein-dependent regulation of MyD88 activation. Wild type (B6) and MyD88-KO macrophages were infected as indicated. Culture supernatants were collected 24 hpi and assayed for indicated cytokines. Results shown (mean and SD; *n* = 3 per group) are representative of three independent experiments. ANOVA (*<0.033; **<0.002; ***<0.001).

MyD88-deficiency abolished the secretion of IL-10 (**Fig 3**), but MyD88 was similarly required for secretion of IL-6 and TNFα (**Fig 3**). Thus, while MyD88 is required for IL-10 secretion from *S*. *pyogenes* infected macrophages the ability of the M5 protein to selectively drive IL-10 secretion cannot be explained by regulation of MyD88 activation.

### IL-10 is induced downstream of M5 protein-dependent type I IFN-signaling

The recent finding that the ESX-1 type VII secretion system–a major virulence determinant of pathogenic mycobacteria–drives an IL-10 producing macrophage phenotype via type I IFN-signaling [10] prompted us to explore if the streptococcal M protein might similarly exploit type I IFN for this purpose.

**Fig 4:**
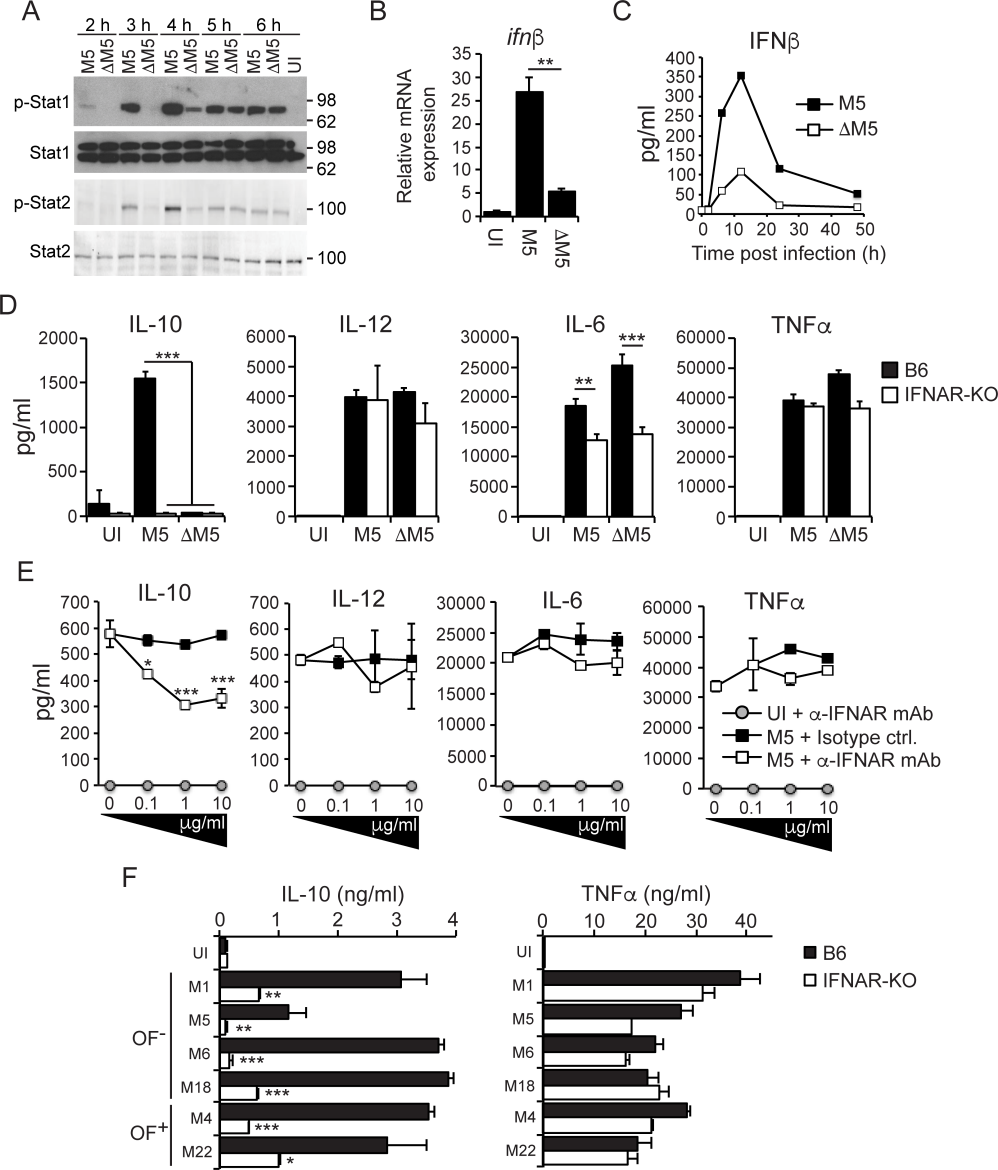
M5 protein-dependent type I IFN-signaling is required for secretion of IL-10. **A)** B6 macrophages were infected with M5 or ΔM5 bacteria, as indicated. Macrophages were lysed at the indicated time points (hours) post infection and analyzed for activation of Stat1 and Stat2 by Western blot. Uninfected (UI) macrophages were analyzed as control. Activated (*i*.*e*. phosphorylated) transcription factors were detected with phospho-specific primary antibodies (indicated with p). Shown is one experiment representative of three. **B)** B6 macrophages were infected with M5 or ΔM5 for 4 hours, and IFNβ transcripts were measured by RTqPCR. mRNA levels are presented as fold-change relative to UI control macrophages. Results shown (mean and SD; *n* = 3 per group) are representative of three independent experiments. **C)** Kinetic analysis of IFNβ secretion from B6 macrophages infected as indicated. Results shown (mean; *n* = 2 per group) are representative of two independent experiments. **D)** B6 and IFNAR-KO macrophages were infected with M5 or ΔM5, or UI, as indicated. Culture supernatants were collected 24 hpi and assayed for indicated cytokines. Results shown (mean and SD; *n* = 3 per group) are representative of three independent experiments. **E)** B6 macrophages were infected with wild type M5 bacteria in the presence of titrated amounts (final concentration indicated) of a neutralizing anti-mouse IFNAR mAb or mouse IgG1 isotype control, as indicated. UI macrophages treated with the anti-IFNAR mAb were analyzed as control. Cytokines were assayed 24 hpi. Results shown (mean and SD; *n* = 3 per group) are representative of two independent experiments. **F)** B6 and IFNAR-KO macrophages were infected with *S*. *pyogenes* strains of six different serotypes (two that are serum opacity factor positive [OF^+^], and four that are OF^-^), as indicated. Cytokines were assayed 24 hpi. Results shown (mean and SD; *n* = 3 per group) are representative of two independent experiments. ANOVA (*<0.033; **<0.002; ***<0.001).

Kinetic analyses of infected macrophages demonstrated that *S*. *pyogenes* promoted the phosphorylation of Stat1 and Stat2 in an M5 protein-dependent manner (**Fig 4A**). Because the type I IFN receptor signals through Stat1/Stat2 heterodimers [21] these findings suggested a key role for the M5 protein in inducing the type I IFN response. Indeed, analyses of IFNβ production at both the mRNA (**Fig 4B**) and protein (**Fig 4C**) levels demonstrated that *S*. *pyogenes* drives type I IFN production largely in an M5 protein-dependent manner.

To test whether IL-10 production was dependent on type I IFN-signaling we infected B6 and type I IFN receptor 1-deficient (IFNAR-KO) macrophages with M5 or ΔM5 *S*. *pyogenes*. Remarkably, M5 protein-mediated secretion of IL-10 was essentially abolished in IFNAR-KO macrophages, while secretion of the proinflammatory cytokines IL-12 and TNFα was not affected (**Fig 4D**). Similar analysis of IL-6 secretion implied a significant but non-required role for type I IFN-signaling (**Fig 4D**). Infection of B6 macrophages with wild type bacteria in the presence of titrated amounts of a neutralizing anti-IFNAR monoclonal antibody specifically and dose-dependently inhibited secretion of IL-10 (**Fig 4E**). Collectively these data demonstrate that IL-10 is produced downstream of M5 protein-dependent type I IFN-signaling. Furthermore, analysis of B6 and IFNAR-KO macrophages infected with strains of different serotypes (M1, M5, M6, M18, M4 and M22) suggested that the ability to exploit type I IFN-signaling to promote an IL-10 producing macrophage phenotype may be a general property of *S*. *pyogenes* (**Fig 4F**). Interestingly, unlike the situation for IL-10 (**Fig 3**), secretion of IFNβ was not significantly affected by MyD88-deficiency (**S4 Fig**), suggesting that both type I IFN- and MyD88-dependent signaling is required for IL-10 production, but likely via distinct processes.

### *S*. *pyogenes* induces type I IFN production via an active process that requires STING but not cGAS

The finding that the M5 protein is required for type I IFN secretion from infected macrophages made it of interest to further explore the mechanistic basis for how *S*. *pyogenes* induces type I IFN production.

**Fig 5:**
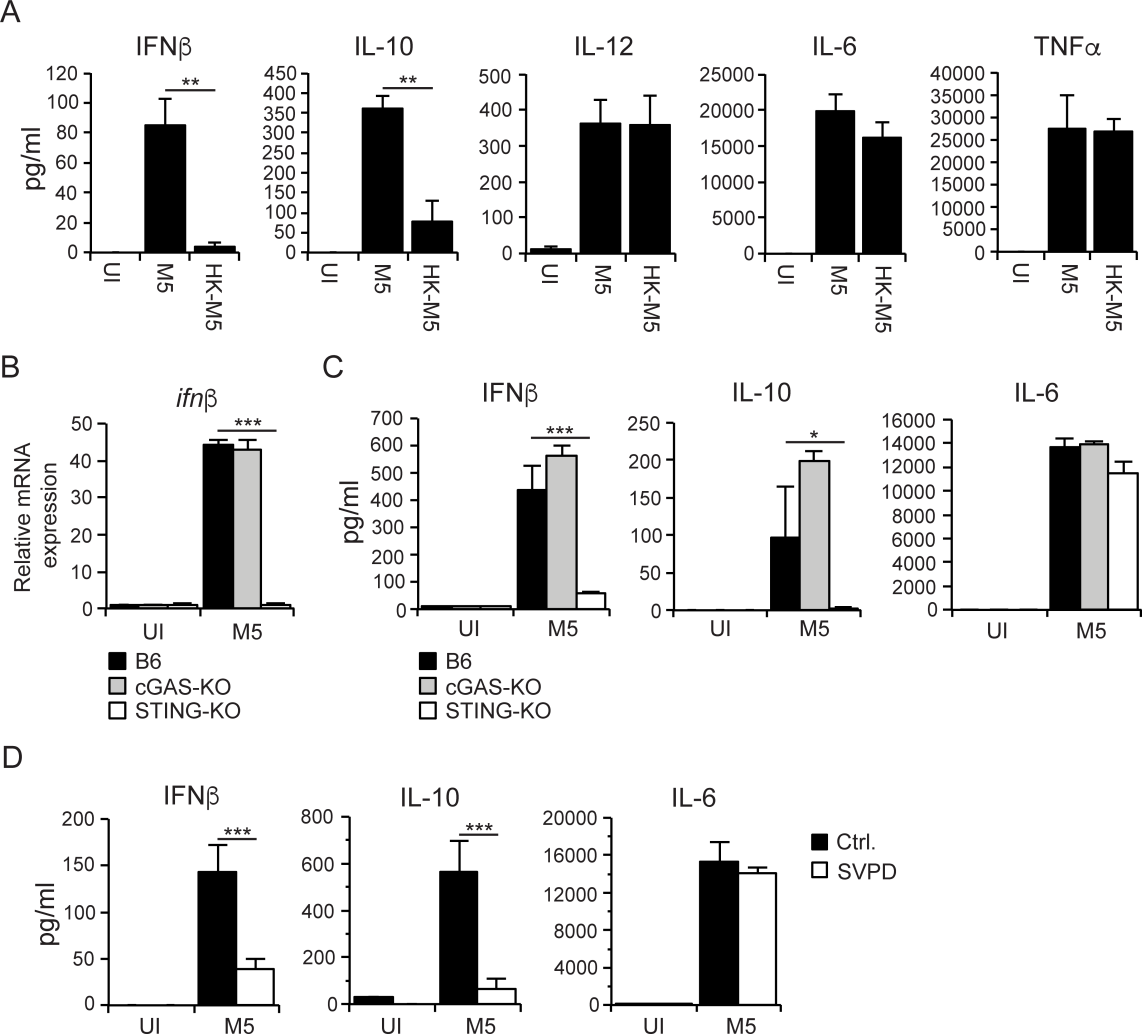
*S*. *pyogenes* induces the STING signaling pathway via an active process that is independent of cGAS. **A)** B6 macrophages were infected with live or heat-killed M5 bacteria, as indicated. Cytokines were assayed 24 hpi. Results shown (mean and SD; *n* = 3 per group) are representative of three independent experiments. **B)** B6, cGAS-KO and STING-KO macrophages were infected with M5 for 4 hours, and IFNβ transcripts were measured by RTqPCR. mRNA levels are presented as fold-change relative to UI B6 macrophages. Results shown (mean and SD; *n* = 3 per group) are representative of three independent experiments. **C)** B6, cGAS-KO and STING-KO macrophages were infected as indicated, and cytokine output was assayed 24 hpi. Results shown (mean and SD; *n* = 3 per group) are representative of three independent experiments. **D)** B6 macrophages were infected with wild type M5 bacteria in the presence (SVPD) or absence (Ctrl.) of snake venom phosphodiesterase (1.3 U/ml final concentration), as indicated. Similarly treated UI macrophages were analyzed as control. Cytokines were assayed 24 hpi. Results shown (mean and SD; *n* = 3 per group) are representative of two independent experiments. ANOVA (*<0.033; **<0.002; ***<0.001).

To assess if induction of type I IFN and downstream IL-10 occurred via an active or passive bacterial mechanism we analyzed cytokine output from B6 macrophages infected with live or heat-killed (HK-M5) wild type bacteria. Interestingly, while secretion of IL-12, IL-6 and TNFα was similar in cells infected with M5 or HK-M5 the induction of IFNβ and IL-10 required viable bacteria (**Fig 5A**), suggesting that *S*. *pyogenes* drives the type I IFN response via an active process that is distinct from how the proinflammatory cytokines IL-12, IL-6 and TNFα are induced.

Earlier studies have demonstrated that *S*. *pyogenes* induces type I IFN in macrophages in a STING-, TBK1- and IRF3-dependent manner [13]. Since it has not previously been determined if the type I IFN response in *S*. *pyogenes* infected macrophages requires the cyclic GMP-AMP synthase (cGAS) for activation of STING we employed cGAS- and STING-deficient macrophages in our system. As expected, STING was required for production of IFNβ, as measured at both the mRNA (**Fig 5B**) and protein (**Fig 5C**) levels. In agreement with the finding that IL-10 production requires M protein-dependent type I IFN-signaling (**Fig 4D**) STING-KO macrophages were unable to produce IL-10 in response to *S*. *pyogenes* infection, whereas IL-6 secretion was essentially unaffected (**Fig 5C**); the apparent paradox that secretion of IL-6 was significantly affected in IFNAR-KO (**Fig 4D**) but not in STING-KO (**Fig 5C**) macrophages might be explained by the former representing the only experimental condition used where type I IFN-signaling is completely absent. Addition of snake venom phosphodiesterase (SVPD)–which cleaves cyclic dinucleotides of both host and bacterial origin [22]–to M5 infected B6 macrophages similarly reduced the secretion of IFNβ and IL-10 without significantly affecting that of IL-6 (**Fig 5D**), implying a role for cyclic dinucleotides in activating STING in our system. Importantly, however, production of IFNβ and downstream IL-10 did not require cGAS (**Fig 5B and 5C**), demonstrating that STING is activated independently of cGAS in *S*. *pyogenes* infected macrophages.

### No detectable release of DNA into the cytosol of *S*. *pyogenes* infected macrophages

Previous work has suggested that *S*. *pyogenes* genomic DNA is responsible for triggering activation of the STING pathway in macrophages [13]. This work, however, was based on stimulation of macrophages with purified *S*. *pyogenes* DNA or sonicated extracts of the bacterium, and did not evaluate the role for DNA during physiological infection. Although cGAS-independent activation of STING has been reported to mediate the immunogenicity of DNA vaccines [23], recent advances in the field suggest that cGAS may be the sole DNA-sensor capable of inducing the STING pathway [24–26]. Our findings (**Fig 5B and 5C**) therefore argue against DNA being the trigger for this pathway in *S*. *pyogenes* infected macrophages. Importantly, the IFNβ response to transfected double stranded DNA was completely abrogated in cGAS-KO macrophages (**S5 Fig**), confirming that these cells are indeed unable to initiate the STING pathway in response to DNA.

**Fig 6:**
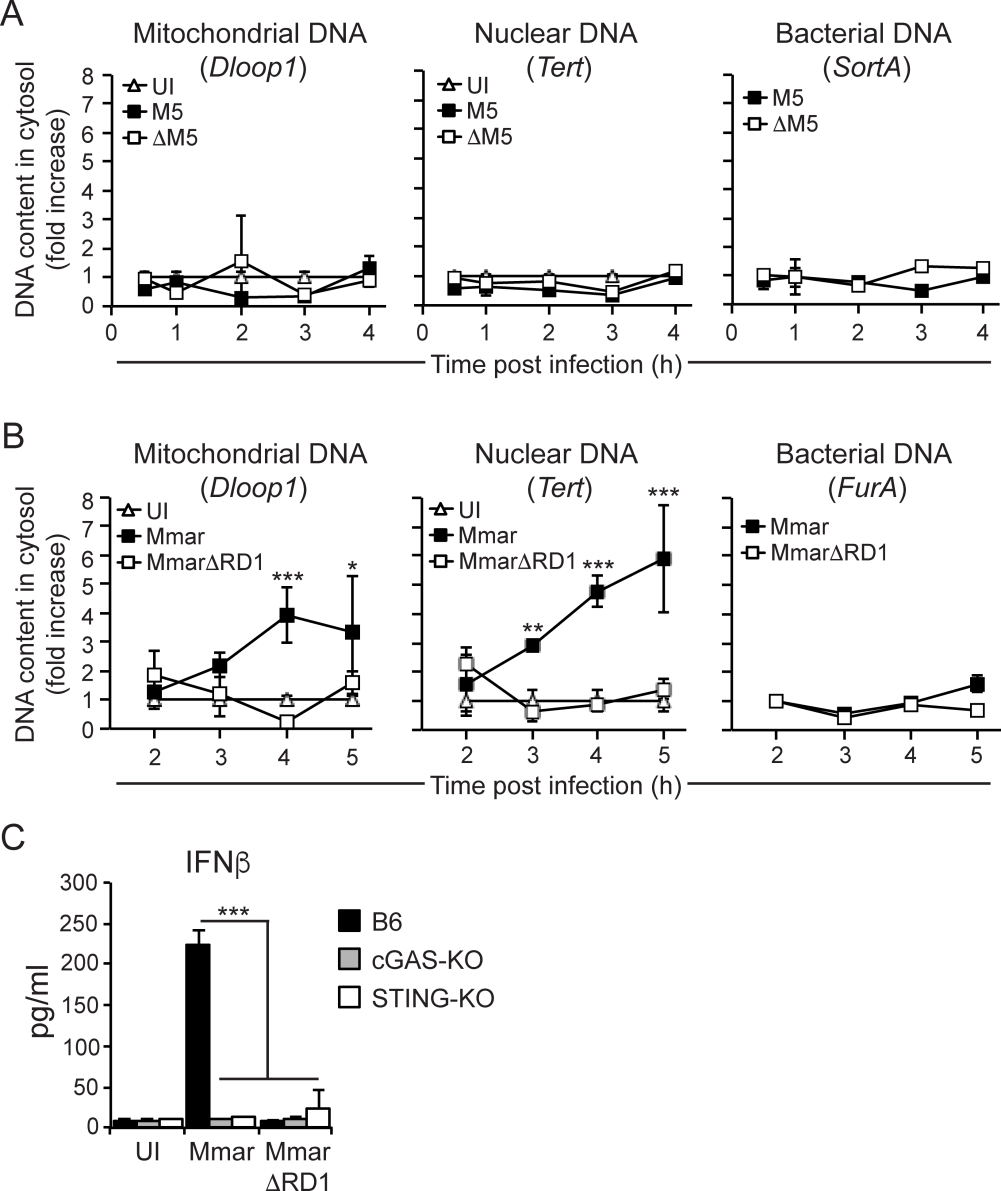
*S*. *pyogenes* does not cause detectable release of DNA of any origin into the cytosol of infected macrophages. **A)** B6 macrophages were infected with M5 or ΔM5, and UI macrophages served as control. At the indicated time points post infection the levels of mitochondrial (*Dloop1*), nuclear (*Tert*) or *S*. *pyogenes* (*SortA*) DNA in the cytosolic compartment was analyzed by qPCR as described in materials and methods. **B)** Same analysis as A) above, but in macrophages infected with wild type *M*. *marinum* (Mmar) or an isogenic ESX-1-deficient mutant (MmarΔRD1). *FurA* was measured to determine the level of *M*. *marinum* DNA in the cytosolic compartment of infected macrophages. For A) and B) the results shown (mean and SD; *n* = 3 per group) are representative of three independent experiments. **C)** B6, cGAS-KO and STING-KO macrophages were infected with Mmar or MmarΔRD1 as indicated, and cytokine output was assayed 24 hpi. Results shown (mean and SD; *n* = 3 per group) are representative of two independent experiments. ANOVA (*<0.033; **<0.002; ***<0.001).

To directly investigate if *S*. *pyogenes* causes release of DNA into the cytosol we purified the cytosolic fraction of infected macrophages and performed quantitative PCR (qPCR) analyses of genes encoded by genomic bacterial (*SortA*) DNA, as well as by mitochondrial (*Dloop1*) and nuclear (*Tert*) host DNA. Kinetic analysis–including the time frame relevant for induction of the type I IFN response (**Fig 4A and 4B**)–of B6 macrophages infected with M5 or ΔM5 bacteria indicated that neither strain caused release of measurable amounts of DNA of any origin into the cytosol (**Fig 6A**). In contrast, *SortA* was readily detectable in the cytosolic fraction of macrophages transfected with purified genomic *S*. *pyogenes* DNA (**S6 Fig**), demonstrating that our experimental approach does allow the detection of *S*. *pyogenes* DNA.

To obtain a positive control for the detection of host DNA in the cytosolic compartment we took advantage of pathogenic mycobacteria. *Mycobacterium tuberculosis* activates the STING pathway in an ESX-1- and cGAS-dependent manner [27, 28], and recent evidence suggests that ESX-1 may activate cGAS indirectly by causing the release of mitochondrial and nuclear, but not bacterial, DNA into the cytosol of infected macrophages [29]. Using the *Mycobacterium marinum* model system we found that infection with wild type bacilli (Mmar), but not with an isogenic ESX-1-deficient mutant (MmarΔRD1), induced significant release of mitochondrial and nuclear DNA into the macrophage cytosol (**Fig 6B**), and that *M*. *marinum* activated the type I IFN response in an ESX-1- and cGAS-dependent manner (**Fig 6C**). These control experiments verify that we are able to detect infection-induced release of mitochondrial and nuclear DNA into the cytosol, further supporting the interpretation that *S*. *pyogenes* does not mobilize DNA into the macrophage cytosol (**Fig 6A**), and activates the STING signaling pathway in a DNA-independent manner.

## Discussion

*In vivo* studies have demonstrated a critical role for macrophages in controlling *S*. *pyogenes* growth and dissemination [5, 6, 30], and depletion of macrophages in BALB/c mice renders these animals highly susceptible to invasive *S*. *pyogenes* infection [6]. However, in addition to their host-protective role in eliminating *S*. *pyogenes*, macrophages shape the ensuing immune response by producing cytokines and chemokines, and unregulated activation of these cells may contribute to host-detrimental inflammation [5, 7]. Thus, macrophages play important but dichotomous roles suggesting the potential benefit for *S*. *pyogenes* to harness the macrophage inflammatory response, as reported here.

Recent data have suggested that stimulation of primed macrophages with purified M proteins may promote inflammasome activation and secretion of IL-1β [31], which together with IL-6 and TNFα is implicated as a key mediator of sepsis. However, purified M proteins did not drive NFκB activation [31], although a previous study had suggested that possibility [32]. Our findings that secretion of IL-6 and TNFα from infected macrophages requires MyD88 but not bacterial viability and M protein expression are consistent with the idea that NFκB is activated by pathogen-associated molecular patterns (PAMPs) in an M protein-independent fashion. MyD88-deficiency may partially affect IFNβ secretion during the early macrophage response to *S*. *pyogenes* infection [13], but we obtained similar levels of this cytokine from B6 and MyD88-KO macrophages already at 24 hours post infection. The finding that MyD88-KO macrophages are unable to secrete IL-10 (as well as IL-6 and TNFα) in response to *S*. *pyogenes* infection despite an almost normal type I IFN response suggests that both MyD88 and type I IFN are required to drive IL-10, but implies that they might do so via distinct processes where the M protein affects only the latter.

Interestingly, extracellular streptococcal NADase specifically suppresses the release of IL-1β from infected macrophages [33], providing a defined strategy by which *S*. *pyogenes* may modulate the inflammatory potential of infected macrophages. Moreover, global gene expression analysis of infected macrophages has shown that *S*. *pyogenes* induces a phenotype encompassing features of both classically and alternatively activated macrophages [8]. Indeed, infection caused upregulation of IL-6, TNFα and IL-1β, typically associated with classical activation, as well as of arginase and IL-10, which is linked to regulatory macrophages and may have profound suppressive effects on both innate and adaptive responses [8]. Our findings confirm that *S*. *pyogenes* infection promotes production of both proinflammatory cytokines and IL-10, and remarkably, identify a key role for the M5 protein in driving the IL-10 producing macrophage phenotype while not affecting the output of proinflammatory cytokines. Moreover, our data demonstrate that IL-10 is produced downstream of M protein-dependent activation of the STING pathway and type I IFN-signaling. Intriguingly, the ESX-1 type VII secretion systems of both *M*. *tuberculosis* and *M*. *marinum*–two pathogens evolutionarily distant from *S*. *pyogenes*–similarly exploit type I IFN-signaling to drive an IL-10 producing macrophage phenotype [10], implying that this strategy might be of broad relevance in bacterial pathogenesis.

It is also interesting to note that the M5 protein promotes Treg polarization upon activation of naïve human T cells by anti-CD3/CD28 treatment *in vitro* [34], suggesting that M proteins might induce a regulatory phenotype of both macrophages and T cells. However, Treg polarization was specifically dependent on interaction between the C-repeat region and CD46 [34], which is not expressed on mouse hematopoietic cells [35], suggesting a different mechanistic basis for induction of regulatory T cells. Furthermore, IL-10 production in our infection model was disrupted by deletions of both the B- and C-repeat regions, suggesting the involvement of distinct functional surfaces in the M5 protein for driving IL-10 production in macrophages and T cells, respectively.

The STING signaling pathway is a main pathway for induction of type I IFN in response to bacterial infection [36]. While several eukaryotic cytoplasmic DNA receptors have been identified cGAS is now thought to play a critical and non-redundant role for induction of STING-dependent type I IFN production in response to cytoplasmic DNA [24–26]. Binding of double stranded DNA of any origin may activate cGAS to generate a specific eukaryotic cyclic dinucleotide (2´,3´-cyclic GMP-AMP) that acts as a second messenger to activate STING [26]. STING subsequently recruits TBK1 to phosphorylate IRF3, which translocates into the nucleus and initiates the transcription of type I IFN [26]. The role for type I IFN in bacterial infection is complex and may differ dependent on etiological agent and infection model. For example, while type I IFN promotes infection with *M*. *tuberculosis* [37–39] and *Listeria monocytogenes* [40–42], it conversely controls infection with *Streptococcus agalactiae* [43, 44]. Interestingly, studies in a *S*. *pyogenes* infection model for lethal subcutaneous cellulitis have demonstrated that type I IFN-signaling promotes host survival by curbing the inflammatory response without impacting on bacterial load [13, 14]; these findings indicate immune-suppressive functions for the type I IFN response in *S*. *pyogenes* infection, and suggest a selective pressure on *S*. *pyogenes* to induce type I IFN in order to ensure host survival. Indeed, our results suggest that *S*. *pyogenes* has evolved to activate the STING pathway via an active and M protein-dependent mechanism that is independent of cGAS and cytosolic sensing of DNA. *S*. *pyogenes* carries a gene encoding a cyclic diadenylate monophosphate (c-di-AMP) synthase [45] and we hypothesize that *S*. *pyogenes*, similarly to *L*. *monocytogenes* [46], might secrete c-di-AMP to activate STING directly. This idea is consistent with our finding that addition of SVPD significantly reduced the secretion of IFNβ, suggesting that the enzyme reaches the cytosol of *S*. *pyogenes* infected macrophages to degrade cyclic dinucleotides required for STING activation; we speculate that streptolysin O (SLO)-mediated membrane permeabilization might facilitate cytosolic access of the SVPD.

The mechanism by which the M protein promotes STING-dependent type I IFN production remains an open question. We considered the possibility that the M protein might facilitate translocation of bacterial cyclic dinucleotides into the host cell cytosol via so-called cytolysin-dependent translocation [47]. However, previous work has demonstrated that induction of type I IFN-signaling in *S*. *pyogenes* infected macrophages is independent of the pore-forming toxins SLO and streptolysin S (SLS) [48], speaking against this possibility and also suggesting that activation of STING might not require host membrane permeabilization. In contrast, other pathogens on the emerging list of bacteria that activate the STING/TBK1/IRF3 signaling axis rely on their ability to permeabilize host membranes, and most require cGAS to activate STING [26]. Collectively, this situation implies unique features to how *S*. *pyogenes* activates the STING pathway in macrophages. While the macrophage-modulating function of the M protein did not correlate with bacterial uptake into macrophages, we speculate that the M protein might affect intracellular trafficking of the bacteria [49] and thereby the extent to which bacterial cyclic dinucleotides interact with the endoplasmic reticulum-localized STING. It will be of great interest to explore this hypothesis and the upstream mechanism by which *S*. *pyogenes* activates STING to regulate macrophage functionality, and to understand its role in pathogenesis.

## Materials and methods

### Ethical statement

All animal care and use adhered to the Swedish animal welfare laws, and to the guidelines set by the Swedish Department of Agriculture (Act 1988:534). These studies were approved by the Malmö/Lund Ethical Board for Animal Research (permit number M9-13). Blood was donated by healthy volunteers that provided oral informed consent (in agreement with the requirements at Lund University), which was documented in laboratory journals.

### Bacterial strains

*S*. *pyogenes* M5 Manfredo is a wild type strain originally isolated from a patient with rheumatic fever [50]. The M5-negative mutant (ΔM5), lacking the entire *emm5*-gene, as well as the transcomplemented strain (ΔM5/pM5) have been described previously [51, 52]. Mutants ΔN1, ΔN2, ΔB, ΔC have in-frame deletions in the *emm5*-gene corresponding to amino acid residues 11–59, 62–110, 118–210 and 215–315 in the mature M5 protein and have been previously characterized [15, 16]. *S*. *pyogenes* strains SF370 (M1), JRS4 (M6), 87–282 (M18), AP4 (M4) and AL168 (M22) were kindly provided by Gunnar Lindahl (Lund University, Sweden). Streptococci were grown in Todd-Hewitt broth supplemented with 0.2% yeast extract (THY) at 37°C in 5% CO_2_ without shaking.

The wild type *M*. *marinum* M-strain and its isogenic deletion mutant lacking the ESX-1-encoding RD1-locus were grown in Middlebrook 7H9 broth (Difco) as previously described [10].

### Flow cytometry analysis of M5 protein surface expression

Over night cultures of bacteria were washed twice and resuspended to 2 x 10^8^ CFU/ml in PBS containing 0.05% Tween-20 (PBST), and incubated for one hour at room temperature with a rabbit antiserum (diluted 500-fold in PBST) specific for the N-terminal region of the M5 protein [16] or a similarly diluted pre-immune serum. Bacteria were washed twice in PBST before incubation with Alexa Fluor 594-conjugated donkey anti-rabbit IgG (Thermo Scientific; diluted 500-fold in PBST) for 30 minutes at room temperature. Bacteria were then washed twice in PBST, fixed in 4% paraformaldehyde (PFA), and run on an Accuri C6 flow cytometer (BD Biosciences). Data was analyzed using the FlowJo software (Tree Star Inc.).

### Generation of bone marrow-derived mouse macrophages and human monocyte-derived macrophages

Bone marrow-derived macrophages were generated from wild type C57BL/6 (B6; BMC Animal Facility, Lund University, Sweden), *ifnar1*^-/-^ (IFNAR-KO; BMC Animal Facility, Lund University, Sweden), *myd88*^-/-^ (MyD88-KO), *Tmem173*^gt^ (STING-KO) and cGAS CRISPR knock-out (cGAS-KO) mice as previously described in detail [53] using BMM-medium (RPMI-1640; Invitrogen) supplemented with 1% L-glutamine, 10 mM Hepes (Sigma), 10% (vol/vol) heat-inactivated fetal calf serum (Sigma; endotoxin ≤0.2 EU/ml), 50 U/ml penicillin G (Gibco), 50 μg/ml streptomycin (Gibco), and 15% (vol/vol) M-CSF-containing supernatant from 3T3-CSF cells. Bone marrow from MyD88-KO, and STING-KO and cGAS-KO mice was kindly provided by Catharina Svanborg (Lund University, Sweden) and Russell Vance (UC Berkeley, USA), respectively. Human monocyte-derived macrophages were generated from freshly drawn human blood, using EDTA as anticoagulant, as previously described [54].

### Macrophage infections

Macrophage infections with *S*. *pyogenes* were performed as previously described [17]. Briefly, overnight cultures of bacteria were washed twice with DPBS (Invitrogen) and resuspended to the appropriate concentration in serum-free Opti-MEM^®^ (Invitrogen). Macrophages were washed twice with warm DPBS and infected at MOI = 40 (unless otherwise specified in the figure legend), and incubated at 37°C in 5% CO_2_. One hour post infection 100 U/ml of Pen/Strep (Gibco) was added to kill off extracellular bacteria. Infections were then incubated (37°C in 5% CO_2_) for the indicated time. Infections with *M*. *marinum* (MOI = 5) were performed as previously described in detail [53].

For analysis of bacterial uptake, infected macrophages were lyzed with 0.1% Triton X-100 (Sigma) for 10 minutes at room temperature at 2 hours post infection, and serial dilutions were plated on blood agar plates for CFU analysis.

### Analyses of cytokine secretion and transcription factor activation

Supernatants from infected macrophages were analyzed by ELISA for production of mouse IL-10, IL-12p40, IL-6 and TNFα using Ready-Set-Go Kits (eBioscience), and mouse IFNβ was measured using the legend max ELISA kit from BioLegend. Human IL-10 and IL-6 were analyzed using BD OptEIA kits (Becton Dickinson).

To compare the cytokine response against live and dead bacteria, respectively, *S*. *pyogenes* suspensions were heat-killed at 65°C for 30 minutes, washed in DPBS and resuspended in Opti-MEM. Killing of bacteria was confirmed experimentally by plating the heat-treated suspensions on blood agar plates for CFU analysis.

To assess the effect of fibrinogen and albumin on cytokine secretion, bacteria were suspended in Opti-MEM supplemented with the indicated final concentration of Fragment D (Hyphen Biomed) or serum albumin (Sigma) before infection of macrophages. The effect of pharmacological blockade of the type I IFN receptor was similarly investigated using a neutralizing anti-mouse IFNAR mAb (MAR1-5A3; BioXCell) or equal amounts of mouse IgG1 isotype control (MOPC-21; BioXCell), and the effect of enzymatic degradation of cyclic dinucleotides was assessed by addition of SVPD (Sigma; 1.3 U/ml final concentration).

For analysis of activation of intracellular signaling pathways, macrophages were put on ice at the indicated times post infection and lyzed with Nonidet-P40-based lysis buffer (1% NP40 [Biochemika]; 150 mM NaCl [Sigma]; 50 mM Tris-base [Sigma], pH 8; 1x Complete EDTA-free protease inhibitor cocktail [Roche]; 1x PhosphoSTOP Easy [Roche]). The cell lysates were then separated by SDS-PAGE, using NuPAGE Novex 12% Bis-Tris Protein Gels (Invitrogen). Rabbit antibodies against mouse phospho-Stat1 (p-Tyr701) and Stat1 were from Cell Signaling Technology. Rabbit antibodies against mouse phospho-Stat2 (p-Tyr689) and Stat2 were from Millipore. All antibodies were used according to the manufacturer’s instructions, and detected with secondary donkey anti-rabbit IgG HRP-conjugated F(ab’)_2_ fragments (Jackson ImmunoResearch Laboratories, Inc.). Membranes were developed with Immun-Star (Bio-Rad).

### Reverse-transcription quantitative PCR (RTqPCR) analysis of IFNβ gene expression

RNA was isolated at 4 hours post infection to generate cDNA using the SV total RNA isolation and GoScript reverse transcription systems, respectively, from Promega. Gene expression was measured by qPCR using SsoFAST EvaGreen Supermix (BioRad) on the iQ5 Real-Time PCR Detection System (BioRad), and normalized to the expression of ribosomal 18S RNA. Primer sequences used were as follows: *ifnβ* forward (ATGAGTGGTGGTTGCAGGC) and reverse (TGACCTTTCAAATGCAGTAGATTCA), and 18S rRNA forward (CTTAGAGGGACAAGTGGCG) and reverse (ACGCTGAGCCAGTCAGTGTA).

### Purification of cytosolic fractions and analysis of cytosolic DNA by qPCR

At the indicated times post infection macrophages were washed twice with warm PBS and subsequently dislodged with 1 ml ice cold PBS. Each sample was divided into two aliquots in order to separately prepare whole cell extracts (WCE) and purified cytosolic fractions, respectively. Cells used to prepare WCE were resuspended in 200 μl 50 μM NaOH (Sigma) and boiled for 30 minutes to solubilize the DNA, and neutralization was subsequently achieved by adding 20 μl 1M Tris-HCl (Sigma) pH8. For purification of the cytosolic fraction cells were centrifuged at 200 g for 5 minutes, resuspended in 500 μl lysis buffer (25 μg/ml digitonin [Sigma], 50 mM Hepes [Sigma], 150 mM NaCl [Sigma]) and kept on ice for 10 minutes. Samples were centrifuged at 1000 g for 3 minutes and supernatants were collected, in a step that was repeated three times. Finally the samples were centrifuged at 17000 g for 10 minutes to remove cellular debris and organelles. DNA was extracted from the cytosolic fractions using the GeneJET PCR Purification kit (Thermo Scientific).

DNA content in the WCE and cytosolic fractions was measured by qPCR using the SsoFAST EvaGreen Supermix (BioRad) on the iQ5 Real-Time PCR Detection System (BioRad). Mitochondrial and nuclear DNA were analyzed using primers for *Dloop1* (forward: ATTCTACCATCCTCCGTGAAACC; reverse: TCAGTTTAGCTACCCCCAAGTTTAA) and *Tert* (forward: CTACGTCATGTGTCAAGACCCTCTT; reverse: GCCAGCACGTTTCTCTCGTT), respectively. For each time point, the cycle threshold (C_t_) value obtained for cytosolic DNA was normalized to the corresponding C_t_ for WCE DNA. The ratios obtained for untreated cell samples were used as reference to calculate the relative fold increases in cytosolic DNA content detected in treated samples (*i*.*e*. the ΔΔC_t_ method). For amplification of *S*. *pyogenes* and *M*. *marinum* genomic DNA we used primers against *SortaseA* (forward: TATGGCGCAGGAACGATGAA; reverse: TTTCAAGCGGCGAAAAGAGC) and *FurA* (forward: CGACACCGAAACGATCTACT; reverse: GCCCACCGAGGTAAGTG), respectively. For these bacterial genes the C_t_ value obtained for cytosolic DNA was similarly normalized to the corresponding WCE DNA C_t_, and the ratio obtained for each time point was subsequently normalized to that obtained for mutant infection (ΔM5 and MmarΔRD1 for *S*. *pyogenes* and *M*. *marinum*, respectively) at the first time point of analysis.

As a positive control for the detection of *S*. *pyogenes* DNA in the cytosolic fraction we transfected 10^6^ macrophages with 60 ng purified genomic DNA from the wild type M5 strain using 4 μg Lipofectamine 2000 (Invitrogen). Mock samples were treated with 4 μg Lipofectamine 2000 alone. After 90 minutes cells were harvested and WCE and cytosolic fractions were prepared and analyzed as described above.

### Analyses of the interaction between the M5 protein and fibrinogen Fragment D

The M5 protein was purified as described [51]. Fibrinogen and fibrinogen Fragment D were from American Diagnostica and Hyphen Biomed, respectively. The binding of ^125^I-labeled M5 protein to fibrinogen or fibrinogen Fragment D was analyzed by Western blot and solid phase radioimmunoassay (SPRIA), essentially as described previously [55]. The ability of fibrinogen and fibrinogen Fragment D to inhibit complement deposition onto the bacterial surface was analyzed by flow cytometry, as previously described in detail [15], using a mouse IgG1 monoclonal antibody directed against human C3d (Quidel Corp.) and mouse IgG1 as isotype control (Quidel Corp.).

### Statistical analysis

GraphPad Prism 7 was used to perform statistical analyses. A 2-way ANOVA with Bonferroni’s multiple comparisons test was used to compare multiple groups.

## Supporting information

S1 Fig Titration of multiplicity of infection (MOI). B6 macrophages were infected with wild type M5 or ΔM5 *S*. *pyogenes* at the indicated MOI, or left uninfected (*i*.*e*. MOI = 0). Culture supernatants were collected 24 hpi and assayed for indicated cytokines. Results shown (mean and SD; *n* = 3 per group) are representative of two independent experiments. ANOVA (*<0.033; **<0.002; ***<0.001).
(TIF)

S2 Fig Bacterial uptake in macrophages. B6 macrophages were infected with M5, ΔM5, M5ΔB or M5ΔC as indicated. Extracellular bacteria were killed of by addition of antibiotics at 1 hpi. At 2 hpi macrophages were washed and lyzed to liberate intracellular bacteria. Lysates were serially diluted and plated onto blood agar plates for CFU analysis. Results shown (mean and SD; *n* = 3 per group) are representative of three independent experiments. ANOVA (*<0.033; **<0.002; ***<0.001).
(TIF)

S3 Fig Functional interaction between fibrinogen Fragment D and the M5 protein. **A)** Fibrinogen was separated into it’s α, β and γ chains by SDS-PAGE (left panel) and blotted for Western blot analysis using ^125^I-labelled M5 protein as a probe (right panel). **B)** Microtiter plates were coated with titrated concentrations of fibrinogen or Fragment D, as indicated. 15000 CPM ^125^I-labelled M5 protein was added to each well, and binding is presented as the percent CPM retained in the well after washing. Data (mean and SD; *n* = 3 per group) are representative of two independent experiments. Results from A and B suggest that the M5 protein binds to the γ chain in fibrinogen Fragment D. **C)** Wild type M5 bacteria were incubated in nonimmune human serum for 10 min with or without addition of fibrinogen (1 mg/ml final concentration) or Fragment D (1 mg/ml final concentration), as indicated. Deposition of C3d on the bacterial surface was subsequently analyzed by flow cytometry. Data are presented as C3d-deposition relative to control (serum alone), and are representative of two independent experiments. Of note, analysis with isotype control IgG1 gave a relative value of 0.4% for bacteria incubated in serum alone, demonstrating specificity of the analysis.
(TIF)

S4 Fig *S*. *pyogenes*-induced secretion of IFNβ from macrophages does not require MyD88. B6 and MyD88-KO macrophages were infected as indicated. Culture supernatants were collected 24 hpi and assayed for IFNβ. Results shown (mean and SD; *n* = 3 per group) are representative of three independent experiments. ANOVA (*<0.033; **<0.002; ***<0.001).
(TIF)

S5 Fig Secretion of IFNβ in response to cytosolic double stranded DNA is abolished in cGAS-KO and STING-KO macrophages. Wild type (B6), cGAS-KO and STING-KO macrophages were transfected with dsDNA (370 ng pTEC15 per 1.25x10^5^ cells) using Lipofectamine 2000. Mock control received Lipofectamine 2000 alone. Culture supernatants were collected 15 hours post transfection and assayed for IFNβ by ELISA. Results shown are mean and SD; *n =* 3 per group.
(TIF)

S6 Fig The *Sortase A* (*SortA*) gene is detectible in the cytosolic fraction of macrophages transfected with *S*. *pyogenes* genomic DNA. 60 ng of purified genomic DNA from *S*. *pyogenes* was used to transfect 10^6^ wild type (B6) macrophages. Mock controls received Lipofectamine 2000 alone. Presence of bacterial DNA in the cytosolic fraction was measured by qPCR analysis of *SortA* as described in materials and methods. Results shown (mean and SD; *n =* 3 per group) are representative of two independent experiments.
(TIF)
