# Supplementary figures and images for: Streptococcal M protein promotes IL-10 production by cGAS-independent activation of the STING signaling pathway
Source: PLoS Pathog. 2018 Mar 26;14(3):e1006969. doi: 10.1371/journal.ppat.1006969 (PMC5886698; doi:10.1371/journal.ppat.1006969)

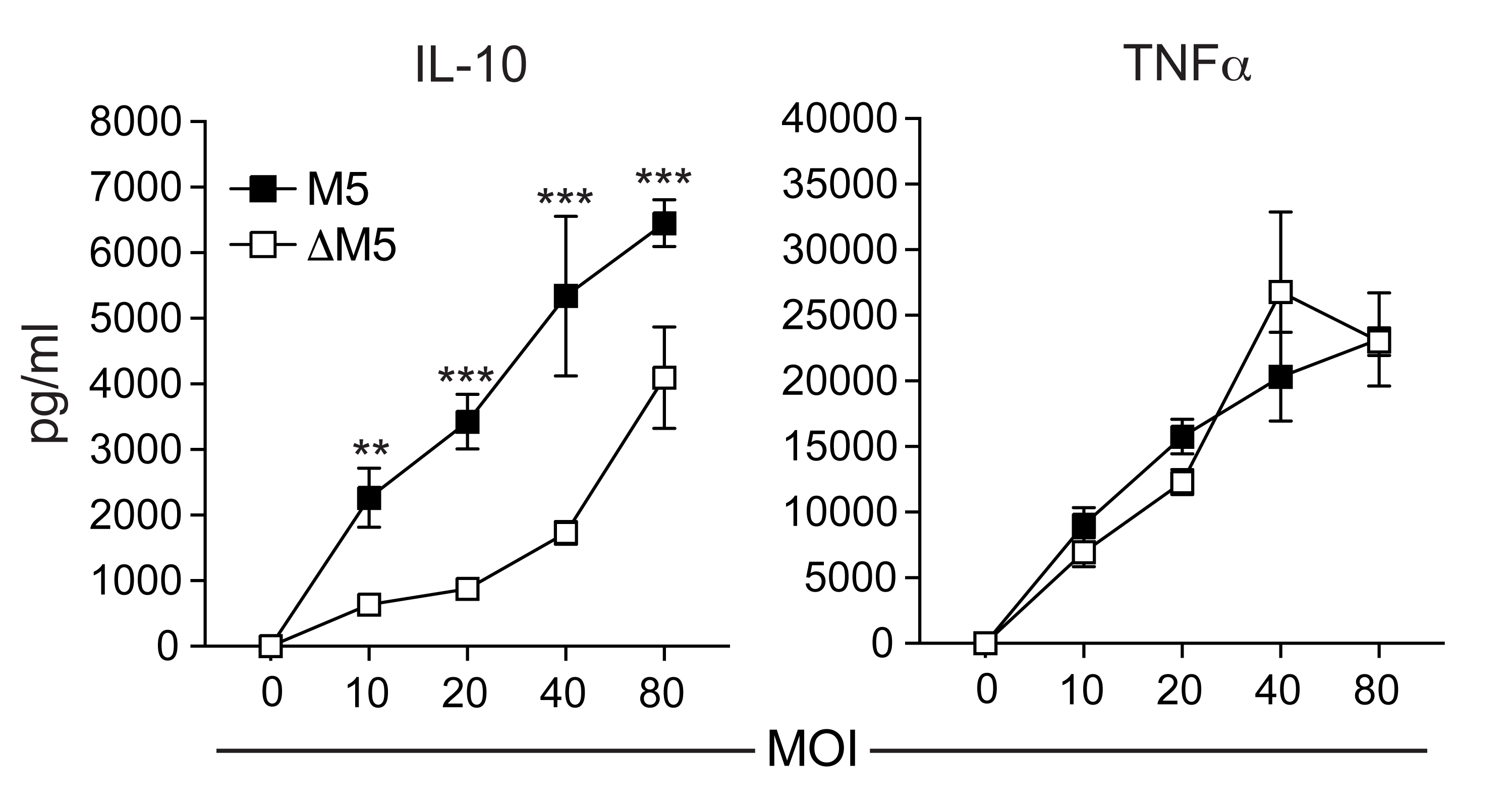

Supplement: S1 Fig — B6 macrophages were infected with wild type M5 or ΔM5 S. pyogenes at the indicated MOI, or left uninfected (i.e. MOI = 0). Culture supernatants were collected 24 hpi and assayed for indicated cytokines. Results shown (mean and SD; n = 3 per group) are representative of two independent experiments. ANOVA (*<0.033; **<0.002; ***<0.001). (TIF) [file ppat.1006969.s001.tif]

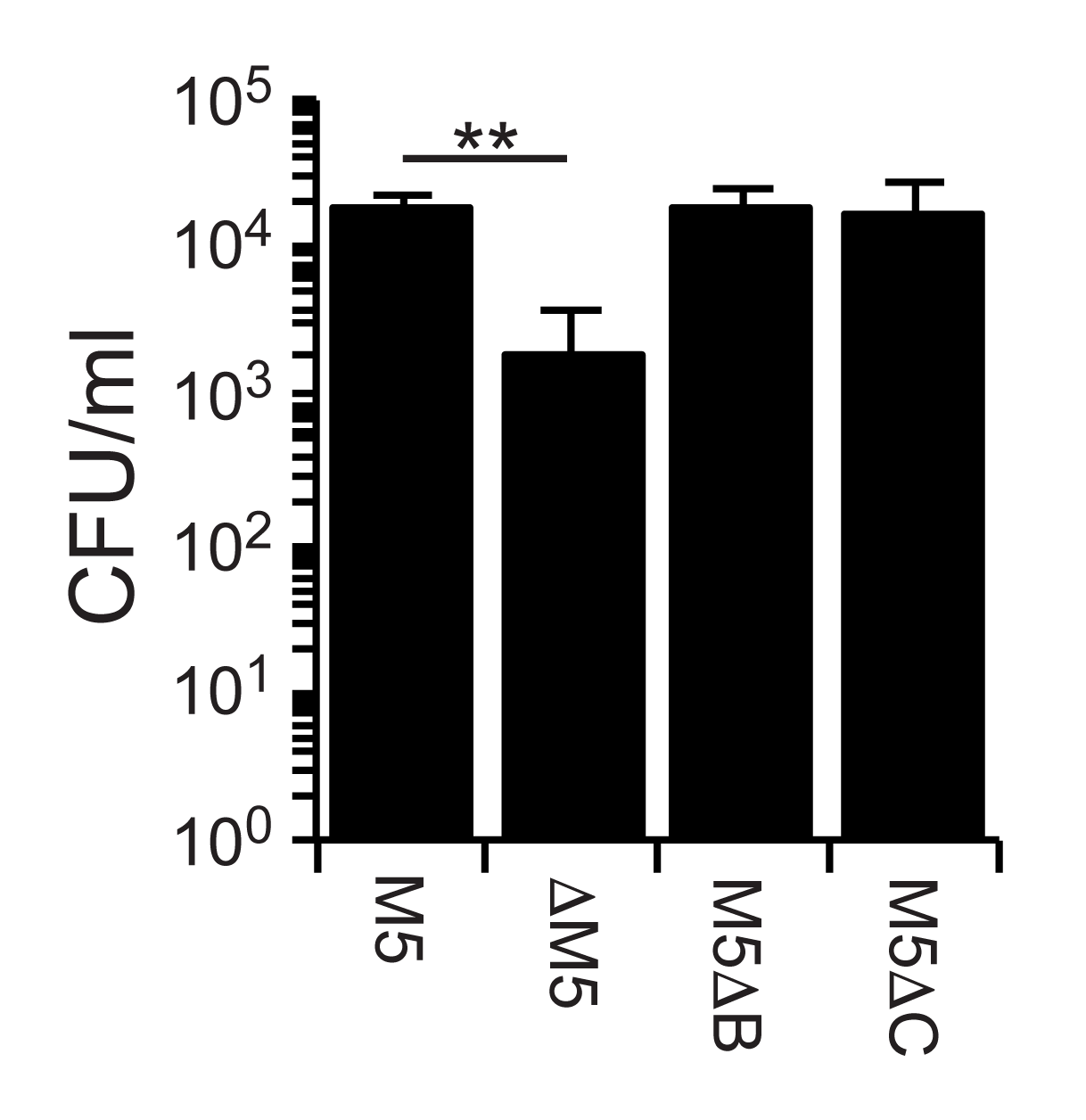

Supplement: S2 Fig — B6 macrophages were infected with M5, ΔM5, M5ΔB or M5ΔC as indicated. Extracellular bacteria were killed of by addition of antibiotics at 1 hpi. At 2 hpi macrophages were washed and lyzed to liberate intracellular bacteria. Lysates were serially diluted and plated onto blood agar plates for CFU analysis. Results shown (mean and SD; n = 3 per group) are representative of three independent experiments. ANOVA (*<0.033; **<0.002; ***<0.001). (TIF) [file ppat.1006969.s002.tif]

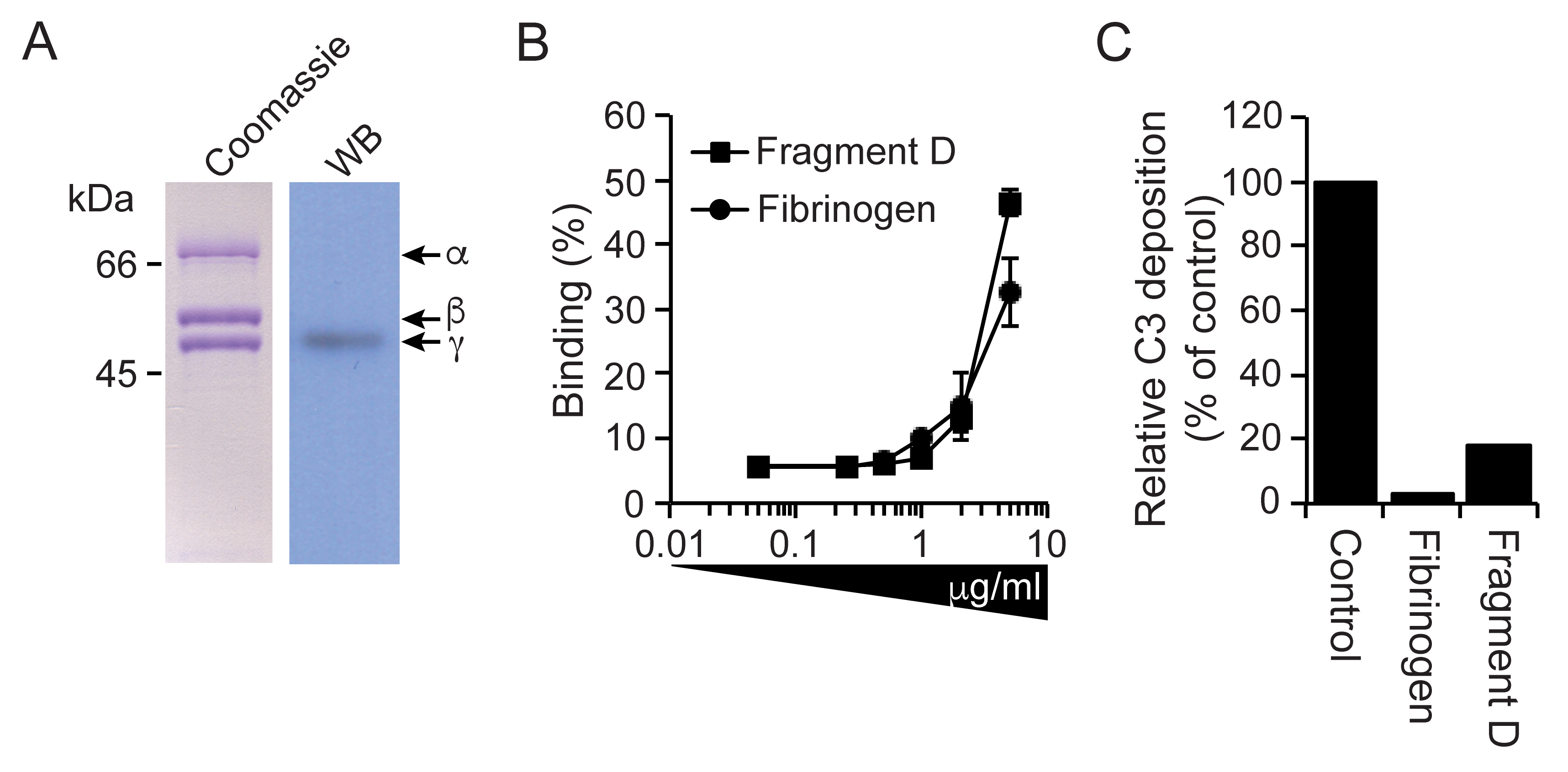

Supplement: S3 Fig — A) Fibrinogen was separated into it’s α, β and γ chains by SDS-PAGE (left panel) and blotted for Western blot analysis using 125I-labelled M5 protein as a probe (right panel). B) Microtiter plates were coated with titrated concentrations of fibrinogen or Fragment D, as indicated. 15000 CPM 125I-labelled M5 protein was added to each well, and binding is presented as the percent CPM retained in the well after washing. Data (mean and SD; n = 3 per group) are representative of two independent experiments. Results from A and B suggest that the M5 protein binds to the γ chain in fibrinogen Fragment D. C) Wild type M5 bacteria were incubated in nonimmune human serum for 10 min with or without addition of fibrinogen (1 mg/ml final concentration) or Fragment D (1 mg/ml final concentration), as indicated. Deposition of C3d on the bacterial surface was subsequently analyzed by flow cytometry. Data are presented as C3d-deposition relative to control (serum alone), and are representative of two independent experiments. Of note, analysis with isotype control IgG1 gave a relative value of 0.4% for bacteria incubated in serum alone, demonstrating specificity of the analysis. (TIF) [file ppat.1006969.s003.tif]

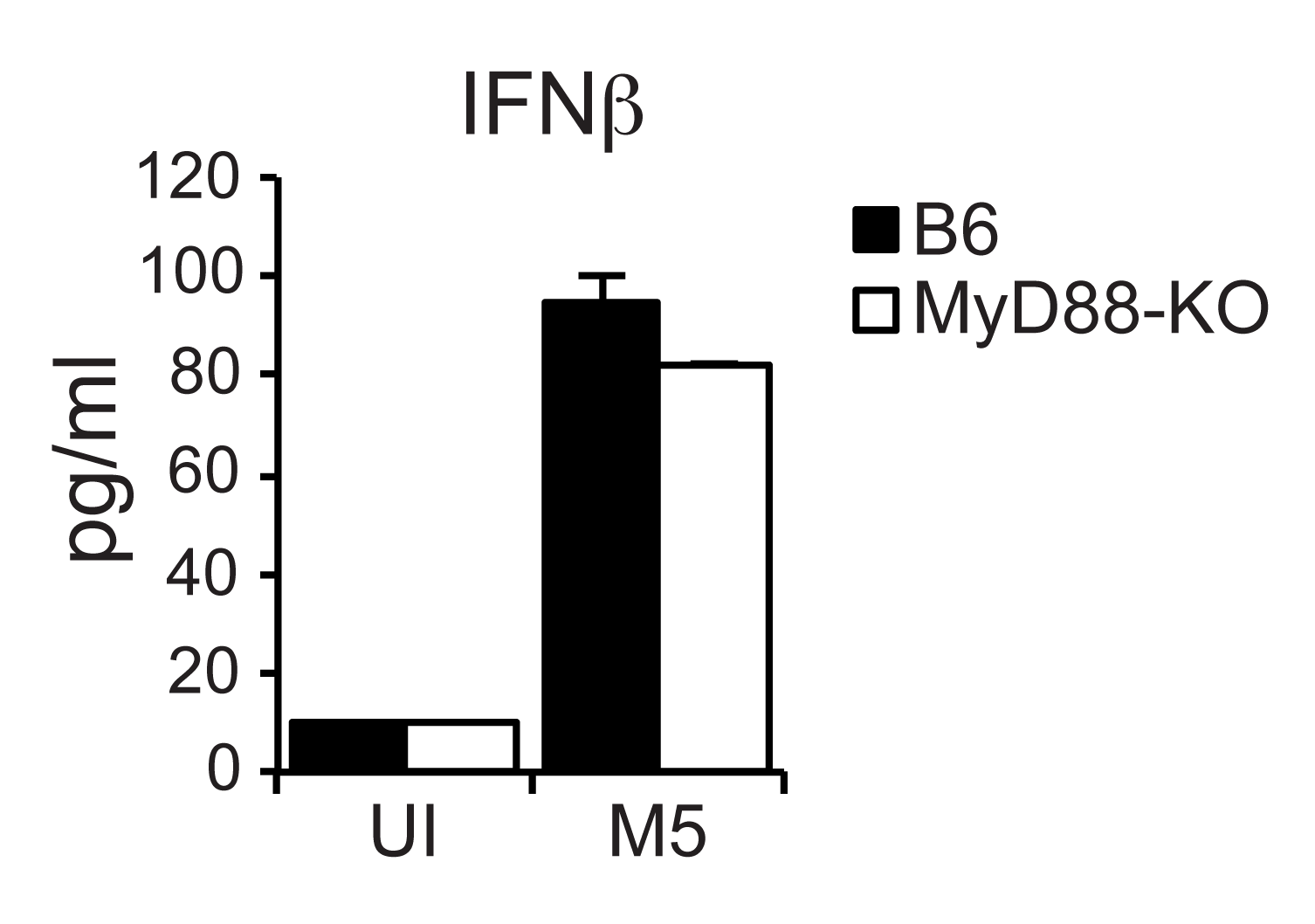

Supplement: S4 Fig — B6 and MyD88-KO macrophages were infected as indicated. Culture supernatants were collected 24 hpi and assayed for IFNβ. Results shown (mean and SD; n = 3 per group) are representative of three independent experiments. ANOVA (*<0.033; **<0.002; ***<0.001). (TIF) [file ppat.1006969.s004.tif]

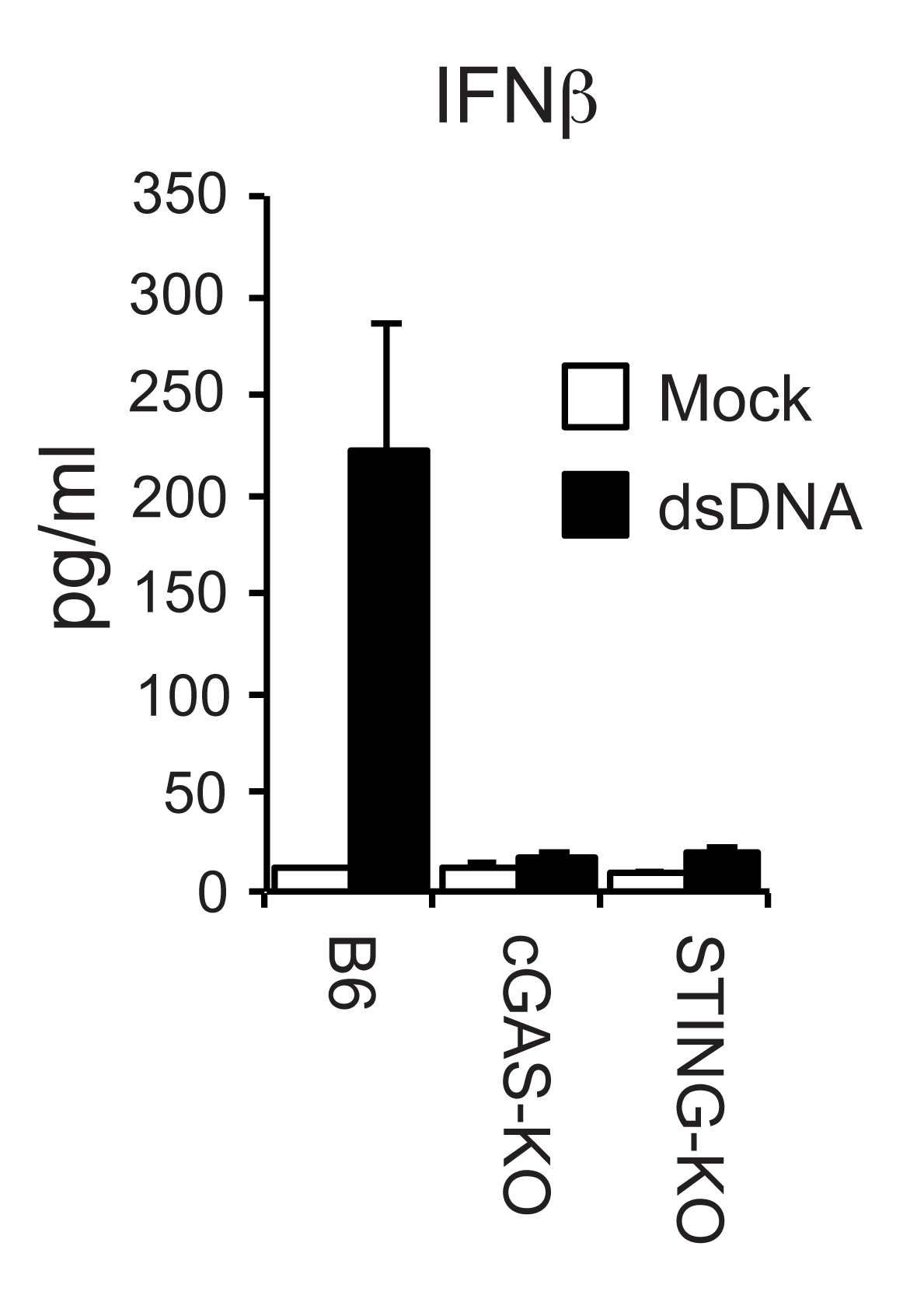

Supplement: S5 Fig — Wild type (B6), cGAS-KO and STING-KO macrophages were transfected with dsDNA (370 ng pTEC15 per 1.25x10^5^ cells) using Lipofectamine 2000. Mock control received Lipofectamine 2000 alone. Culture supernatants were collected 15 hours post transfection and assayed for IFNβ by ELISA. Results shown are mean and SD; n = 3 per group. (TIF) [file ppat.1006969.s005.tif]

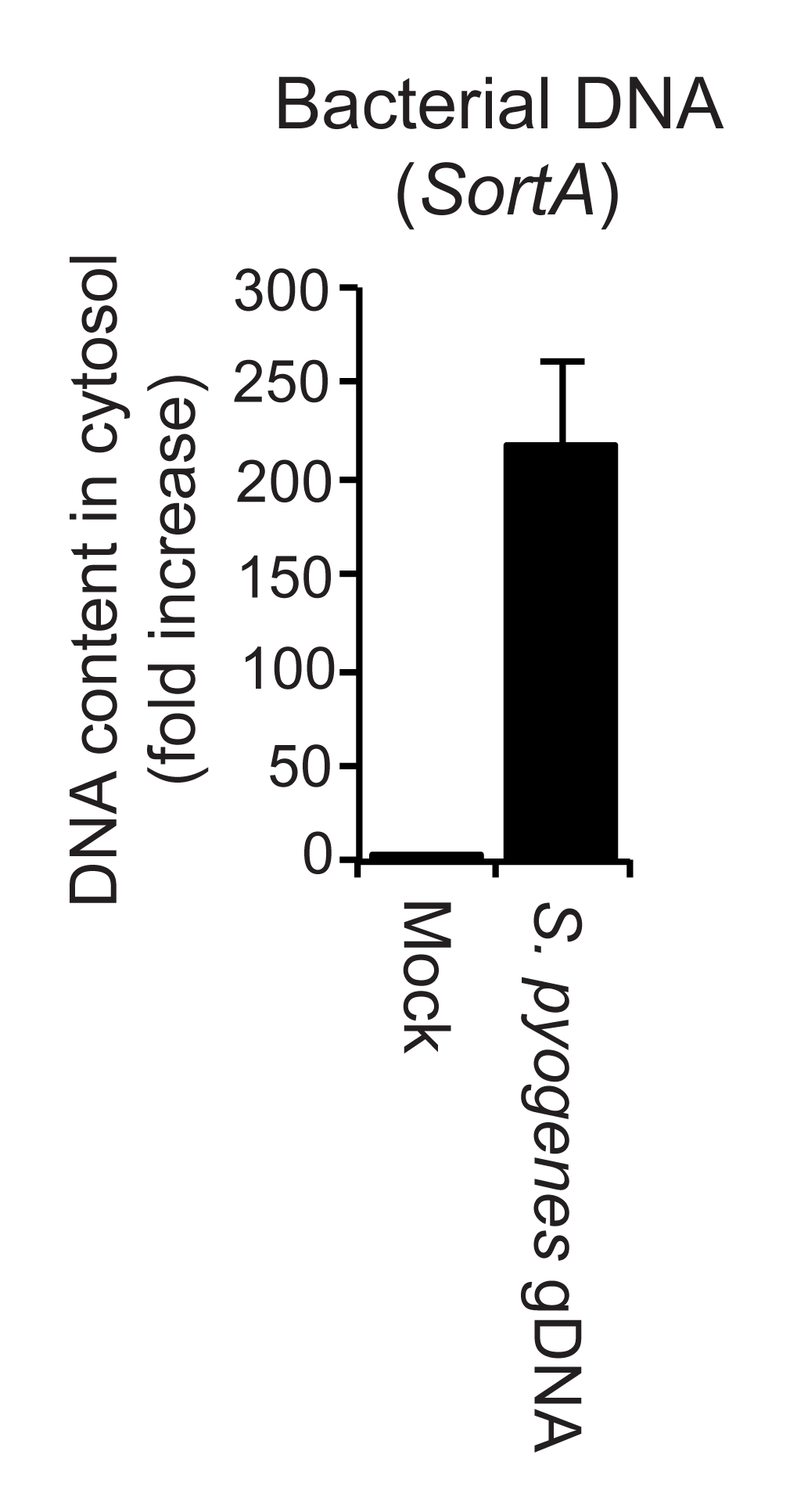

Supplement: S6 Fig — 60 ng of purified genomic DNA from S. pyogenes was used to transfect 106 wild type (B6) macrophages. Mock controls received Lipofectamine 2000 alone. Presence of bacterial DNA in the cytosolic fraction was measured by qPCR analysis of SortA as described in materials and methods. Results shown (mean and SD; n = 3 per group) are representative of two independent experiments. (TIF) [file ppat.1006969.s006.tif]
